# Clinical Predictors of Survival After Palliative Radiotherapy for Glioblastoma in a Real-World Cohort Study

**DOI:** 10.3390/curroncol33060305

**Published:** 2026-05-23

**Authors:** Felix Bock, Guido Hildebrandt, Bernd Frerker, Siyer Roohani, Hannah Lily Fänger, Yvonne Dzierma

**Affiliations:** 1 Department of Radiotherapy and Radiation Oncology, Rostock University Medical Center, 18057 Rostock, Germany; guido.hildebrandt@med.uni-rostock.de (G.H.); bernd.frerker@med.uni-rostock.de (B.F.); hannah.faenger@uni-rostock.de (H.L.F.); yvonne.dzierma@med.uni-rostock.de (Y.D.); 2 Department of Radiotherapy and Radiation Oncology, Hamburg-Eppendorf University Medical Center, 20251 Hamburg, Germany; s.roohani@uke.de

**Keywords:** glioblastoma, palliative radiotherapy, overall survival, early mortality, prognostic factors, patient selection

## Abstract

Background: This study sought to evaluate overall survival (OS) following palliative radiotherapy in glioblastoma and to identify clinically applicable predictors supporting patient-centred treatment decisions, with exploration of early mortality. Methods: This retrospective single-centre study analysed 169 glioblastoma patients treated with palliative radiotherapy between 2010 and 2025. Baseline clinical and treatment variables were assessed. OS calculated from the last day of radiotherapy was estimated using Kaplan–Meier analysis; predictors were analysed using Cox regression. Early mortality (≤30 and ≤60 days) was evaluated using logistic regression. Results: Median age at diagnosis was 75 years, median Karnofsky Performance Status (KPS) was 60%, with 63% of patients < 70%. Impaired mental status, sensorimotor deficits, and steroid use were observed in 47%, 68%, and 86% of patients, respectively. Median OS was 3.5 months. Impaired mental status (HR 2.25), sensorimotor deficits (HR 1.77), steroid use (HR 1.39), multilobar involvement (HR 1.44), and age (HR 1.03) were independently associated with OS, whereas KPS was not. The rate of early mortality at 30 and 60 days was 18% and 31%. Early mortality analysis indicated impaired mental status and steroid use as indicators of very limited survival. Conclusions: Impaired mental status and steroid use identify patients with limited survival, whereas KPS lacks independent prognostic value in this setting.

## Simple Summary

Glioblastoma is an aggressive brain tumour that can be associated with poor performance scores and, consequently, treated with palliative radiotherapy. In this setting, it is unclear who truly benefits from this approach, and further predictors are needed to guide treatment decisions. In this study of 169 patients treated with palliative radiotherapy, survival was generally short, with high early mortality rates. We found that simple clinical features, especially impaired mental status and steroid use, were linked to poorer outcomes, while a commonly used performance score was less informative. These findings suggest that easily assessable clinical parameters can help to identify patients with very limited life expectancy. This could support more realistic discussions with patients and families, avoid unnecessary treatments, and improve patient-centred care. Future research should validate these variables and integrate them into practical decision-support tools.

## 1. Introduction

Glioblastoma is the most prevalent malignant primary brain tumour in adults, with an annual incidence of 3–5 cases per 100,000 individuals globally [1,2]. The highest rates have been reported in developed and high-income regions, such as North America (5.46/100,000) and Western Europe (5.56/100,000) [3]. Several studies have shown a rising incidence in recent decades, with projections estimating 474,000 new cases worldwide by 2045, a 47% increase compared to 2022. This rise is mainly driven by demographic changes [3,4], leading to a growing number of elderly patients facing complex treatment decisions.

The incidence of glioblastoma increases with age, showing its peak between 75 and 84 years [5], rendering glioblastoma predominantly a disease of elderly and often multimorbid patients. Additionally, approximately 50% of patients present with compromised decision-making capacity at the time of diagnosis due to cognitive impairment, behavioural alterations, and communication difficulties [6], which further complicates shared decision-making in routine clinical practice.

Despite multimodal therapy, median OS remains between 12 and 15 months, with a 5-year overall survival rate of <10% [1]. In elderly patients and those with limited performance status, treatment is often restricted to palliative approaches. Therefore, a substantial proportion of patients receive palliative radiotherapy [7,8,9]. In particular, in patients with poor clinical conditions, the benefit of this approach is uncertain, and a subset of patients experiences very limited survival despite treatment [10].

In daily clinical practice, treatment decisions in such settings are complex and frequently made in the context of shared decision-making processes involving not only patients but also their relatives. In some cases, palliative radiotherapy is initiated despite limited functional status and high symptomatic burden, reflecting patient- or family-driven preferences along with the hope to achieve some improvement of the clinical status. Additionally, patients are sometimes referred for radiotherapy shortly after surgical intervention, allowing limited time for comprehensive clinical reassessment. Consequently, palliative radiotherapy is administered to patients with unfavourable prognostic profiles.

To improve treatment decisions and better align therapeutic interventions with realistic patient outcomes, it is necessary to identify reliable and easily assessable predictors associated with limited survival to support shared decision-making. While established prognostic tools such as Karnofsky Performance Status (KPS) [10] and Recursive Partitioning Analysis (RPA) are widely used to estimate survival in curatively intended treatment, their discriminatory power is limited in purely palliative populations, where a large proportion of patients already present with poor functional status [11,12,13]. This limitation highlights the need for additional, more precise, and readily applicable predictors tailored to this challenging cohort [14].

Consequently, the present study aimed to analyse OS following palliative radiotherapy for glioblastoma in a real-world cohort and to identify clinically applicable predictors of survival. Early mortality at 30 and 60 days was explored as a secondary endpoint to provide additional clinical context for treatment decision-making in patients with very limited life expectancy.

## 2. Materials and Methods

### 2.1. Patients

The clinical data of 169 patients with pathologically confirmed World Health Organization (WHO) grade IV glioblastoma treated with palliative radiotherapy alone between 2010 and 2025 at the Department of Radiotherapy and Radiation Oncology, University Medical Center Rostock, Germany, were retrospectively analysed. Only patients with available baseline clinical data and follow-up information were included in this study.

Baseline characteristics, including age, sex, and KPS, were collected at the initiation of palliative radiotherapy. The RPA class was determined based on age, performance status, mental status, type of resection, and neurological function [12]. Neurological status was documented, including mental status and the presence of sensorimotor deficits. Impaired mental status was defined as impairment in orientation to at least one of the following domains: time, place, situation, or person, as documented in routine clinical assessment. Tumour-related variables included tumour localisation and involvement of more than one lobe (multilobar). O6-methylguanine-DNA methyltransferase (MGMT) promoter methylation status was recorded when available.

### 2.2. Treatment

Radiotherapy was administered utilising 6 MV photons, employing either intensity-modulated radiotherapy (IMRT) or volumetric modulated arc therapy (VMAT) with image guidance, in accordance with institutional protocols. The gross tumour volume (GTV), clinical target volume (CTV), and planning target volume (PTV) were delineated on planning CT scans, incorporating pretreatment imaging. The prescribed total radiation dose was typically 39.0 Gy (average [AVG]) in 3.0 Gy (AVG) fractions to the PTV.

### 2.3. Study Design and Ethics

This retrospective, single-centre analysis utilised prospectively documented routine clinical data. Treatment decisions were made independently of this analysis in accordance with contemporary guidelines and the clinical judgement of the treating physicians.

The study was conducted in accordance with the Declaration of Helsinki and approved by the Institutional Review Board of the University Medical Center Rostock (A2024-0221).

### 2.4. Endpoints and Statistical Analysis

Statistical analyses were performed using R version 4.2 (R-CRAN, 2021). Descriptive statistics were reported as absolute and relative frequencies (percentages) for categorical variables, and as mean, median, and sample size, with percentiles provided where appropriate for continuous variables.

OS was particularly defined as the time from the end of radiotherapy to death by any cause, reflecting post-treatment outcomes relevant for clinical decision-making. Survival curves were estimated using the Kaplan–Meier method. Patients were censored at the last available follow-up if no death was observed.

To identify factors associated with OS, univariate and multivariate Cox proportional hazards regression analyses were performed. Variables that were statistically significant in the univariate analysis or considered clinically relevant were included in the multivariate model. Results are presented as hazard ratios (HRs) with 95% confidence intervals (CIs). To avoid collinearity, Recursive Partitioning Analysis (RPA) classification was not included in the multivariate model.

Early mortality was defined as death within 30 or 60 days after the end of radiotherapy. Multivariate logistic regression analysis was performed to identify factors associated with early mortality. Results are presented as odds ratios (ORs) with 95% confidence intervals (CIs). Given the limited number of events for 30-day mortality, the results of logistic regression analyses are interpreted as exploratory.

All statistical tests were two-sided, and a *p*-value < 0.05 was considered statistically significant.

## 3. Results

### 3.1. Patient, Tumour and Treatment Characteristics

A total of 169 patients were included in this study. The median age at diagnosis was 75 years (range, 49–91 years). The median Karnofsky Performance Status (KPS) was 60% (range, 20–90%), with 63% (106/169) of patients presenting with KPS < 70%. The extent of resection was 14% (24/169) and 47% (80/169) for gross total and subtotal resections, respectively, while 38% (65/169) received biopsy only. The median time span between surgery and the initiation of radiotherapy was 19 days (range, 8–88 days). Recursive partitioning analysis (RPA) classes were distributed as follows: class IV in 14% (24/169), class V in 47% (79/169), and class VI in 39% (66/169). Impaired mental status was present in 47% (80/169) of patients, and sensorimotor deficits were observed in 68% (115/169). Steroid use was documented in 86% (146/169) of patients. MGMT promoter status was methylated in 26% (44/169), unmethylated in 45% (76/169), and unknown in 29% (49/169). Radiotherapy was predominantly delivered using hypofractionated regimens, with a median fraction dose of 3.0 Gy (range 2.0–3.0) and a median total dose of 39.0 Gy (range 3.0–45.0). A total of 95% (161/169) of patients completed the radiotherapy course. Table 1 summarises patient, tumour, and treatment characteristics.

### 3.2. Overall Survival

**Figure 1:**
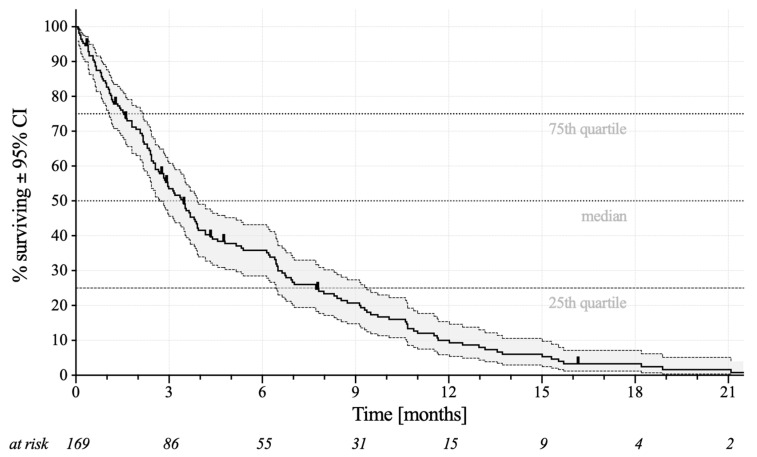
Kaplan–Meier plot for overall survival (OS) following the last day of palliative radiotherapy in patients with glioblastoma. The grey-shaded area represents the 95% confidence interval (CI). Patients at risk at selected time points are displayed below the x-axis.

OS was limited, with 158/169 deaths (93.5%) observed during follow-up. The median OS was 3.5 months (mean: 5.8 months), calculated from the last day of radiation (Figure 1).

In univariate Cox regression analysis, KPS ≥ 70% vs. <70% (HR 0.49, 95% CI 0.35–0.68, *p* < 0.001), patients classified as RPA class VI (HR 1.81, 95% CI 1.18–1.93, *p* = 0.001), impaired mental status (HR 2.70, 95% CI 1.47–3.44, *p* < 0.001), sensorimotor deficits (HR 2.10, 95% CI 1.49–2.96, *p* < 0.001), and steroid use (HR 1.51, 95% CI 1.18–1.93, *p* = 0.001) were significantly associated with a shorter OS. Conversely, age (HR 1.12, 95% CI 0.94–1.33, *p* = 0.22), MGMT promoter status (HR 1.36, 95% CI 0.92–2.01, *p* = 0.13), and multilobar tumour involvement (HR 1.29, 95% CI 0.94–1.76, *p* = 0.12) did not exhibit significant associations with OS in univariate analysis.

**Figure 2:**
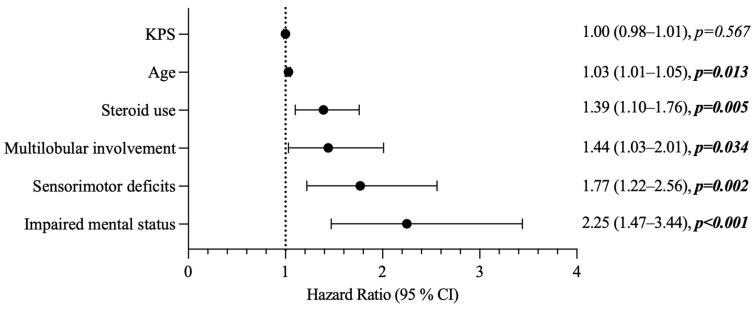
Forest plot of multivariate Cox proportional hazards regression analysis for overall survival. The vertical dashed line indicates the reference value (HR = 1.0).

In multivariate Cox regression including KPS, impaired mental status (HR 2.25, 95% CI 1.47–3.44, *p* < 0.001), sensorimotor deficits (HR 1.77, 95% CI 1.22–2.56, *p* = 0.002), multilobar tumour involvement (HR 1.44, 95% CI 1.03–2.01, *p* = 0.034), steroid use (HR 1.39, 95% CI 1.10–1.76, *p* = 0.005), and age per year (HR 1.03, 95% CI 1.01–1.05, *p* = 0.013) were independently associated with OS. In contrast, KPS did not retain statistical significance in the multivariate model (HR, 1.00; 95% CI, 0.98–1.01; *p* = 0.567). Figure 2 presents the Forest Plot for multivariate Hazard Ratios.

### 3.3. Early Mortality

The mortality rates at 30, 60, and 90 days were 18% (31/169), 31% (53/169), and 49% (82/169), respectively. Mortality at both 30 and 60 days differed according to mental status. Specifically, the 30-day mortality rate was 33.8% (27/80) among patients with impaired mental status, compared to 2.2% (2/89) among those with normal mental status. Similarly, the 60-day mortality rates were 56.2% and 4.5%, respectively.

**Figure 3:**
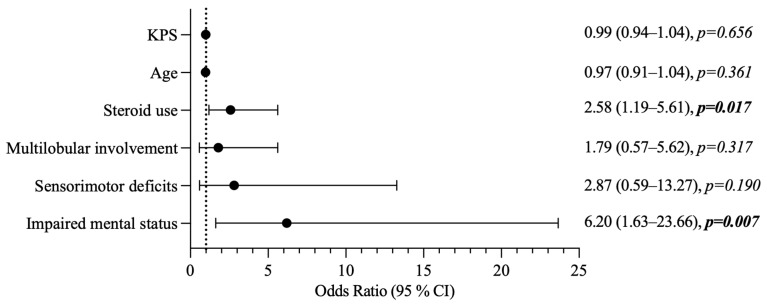
Forest plot of multivariate logistic regression analysis for 60-day mortality. The vertical dashed line indicates the reference value (OR = 1.0).

In exploratory multivariate logistic regression analysis, impaired mental status was consistently associated with 30-day mortality (OR 11.06, *p* = 0.036), although estimates were imprecise. Regarding 60-day mortality, both impaired mental status (OR 6.20, 95% CI 1.63–23.66, *p* = 0.007) and steroid use (OR 2.58, 95% CI 1.19–5.61, *p* = 0.017) were independently associated. Additional variables, including KPS and age, did not demonstrate an independent association with early mortality in multivariate logistic regression analysis. A forest plot of the multivariate logistic regression analysis for 60-day mortality is shown in Figure 3.

## 4. Discussion

In this real-world cohort of glioblastoma patients almost exclusively treated with hypofractionated palliative radiotherapy of 39 Gy in 13 fractions, OS was limited, with a median survival of 3.5 months. In multivariate analysis, impaired mental status, sensorimotor deficits, steroid use, multilobar involvement, and older age were independently associated with worse OS, whereas KPS did not retain independent prognostic significance. In addition, early mortality was high, with 18% and 31% of patients deceased within 30 and 60 days, respectively. Impaired mental status and steroid use were also consistently associated with early mortality, further underscoring their role as clinically relevant indicators of very limited survival. Median follow-up was 142 days.

Our observed overall survival outcomes are consistent with the previously published literature on elderly and frail patients [10,15,16,17,18,19], taking into account that OS was calculated from the last day of radiotherapy. Impaired mental status, steroid use, and sensorimotor deficits likely reflect a more advanced tumour burden with compromised neurological and cognitive function, both of which have been associated with poorer outcomes in glioblastoma patients [20,21]. Consistent with prior studies, cognitive impairment has been identified as an independent prognostic factor for survival [22,23,24,25,26]. However, data specifically addressing its role in purely palliative radiotherapy cohorts are limited. Corticosteroids, nevertheless, are frequently administered following cranial surgery. Their use at the initiation of radiotherapy may therefore partly reflect routine postoperative management rather than disease severity alone and should be interpreted within this broader clinical context, rather than as an isolated decision-making marker. This is further supported by the median time between surgery and the initiation of radiotherapy, which was 19 days in our cohort. Furthermore, corticosteroids may also exert direct adverse effects through immunosuppression and modulation of the tumour microenvironment, which could further influence survival [27].

Notably, although KPS was significantly associated with OS in univariate analysis, it did not retain independent significance in our multivariate models. This contrasts with previous studies in glioblastoma, where KPS has consistently been identified as a solid prognostic factor [11,28] and is a cornerstone of the Radiation Therapy Oncology Group (RTOG) Recursive Partitioning Analysis (RPA) classification, one of the most widely used prognostic tools in glioblastoma. However, these analyses primarily included patients receiving multimodal, mostly curatively intended, therapy, excluding patients older than 70 years and including only a limited number of patients treated in a palliative setting [12,13]. In our cohort, most of the patients presented with poor performance status (median KPS: 60%, range: 20–90%), leaving KPS values clustered in the lower range and thereby reducing variability and prognostic discrimination. Limitations of performance-based scores in selected subgroups have been described before [20] and could explain the lack of multivariate prognostic significance in our study.

Our results show that, in a predominantly low-performance and purely palliative population, common performance-based scores alone may be insufficient in guiding clinical decision-making, while easily assessable parameters, such as impaired mental status and steroid use, may allow the identification of a subgroup of patients with high risk of early death and low likelihood of meaningful benefit from palliative radiotherapy. In clinical practice, our findings are especially relevant, as treatment decisions are often made in close collaboration with relatives in such cohorts. Assessing these parameters will therefore support more realistic discussions regarding survival and help to better align treatment decisions with patient-centred goals.

The strengths of this study include the analysis of a large consecutive real-world cohort treated with a near-uniform radiotherapy regimen, the focus on a purely palliative population, and the evaluation of readily available clinical variables. However, several limitations should be acknowledged. The retrospective design introduces potential selection and information bias. The assessment of mental status and sensorimotor deficits was based on routine clinical documentation rather than standardised instruments, which could have resulted in misclassification. Steroid use was recorded only as a binary variable; more detailed information was not consistently available, and the difference between routine postoperative steroid use and symptom-driven dependence could not be assessed. Additionally, the number of events for early mortality, particularly for 30-day mortality, was limited, which resulted in unstable estimates in multivariate logistic regression. These analyses should therefore be interpreted as exploratory.

In the future, prospective, multi-centre studies are warranted to validate our findings and to integrate simple clinical and neurological parameters into robust prognostic models or decision-support tools, ensuring broader generalizability. Furthermore, the incorporation of short-term patient-reported outcomes is essential to better define the clinical value of palliative radiotherapy and to support patient-centred treatment decisions in patients with very limited life expectancy.

## 5. Conclusions

In this real-world cohort of patients with glioblastoma undergoing palliative radiotherapy alone, overall survival was limited, and early mortality was substantial. Impaired mental status and steroid use were consistently associated with markedly reduced survival, whereas KPS did not retain independent prognostic value in this predominantly low-performance population. These parameters improve prognostic assessment beyond performance-based scores and support shared decision-making, particularly in patients with impaired decision-making capacity. Their integration into routine clinical assessment may help to better align treatment decisions with realistic outcomes and patient-centred goals in a purely palliative setting.

## Figures and Tables

**Table 1:** Patient, tumour, and treatment characteristics.

Characteristics		N	%
Age (years)	Mean, standard deviation	73	±8.7
	Median, range	75	49–91
Age group (years)	<60	17	10
	60–<70	29	17
	70–<80	84	50
	≥80	39	23
Sex	Male	90	53
	Female	79	47
Karnofsky Performance Status (KPS, %)	Mean, standard deviation	57.3	±15.3
	Median, range	60	20–90
KPS group	<70%	106	63
	≥70%	63	37
Surgery	Gross total resection	24	14
	Subtotal resection	80	48
	Biopsy only	65	38
Time between surgery and radiotherapy (days)	Mean, standard deviation	23	±13.9
	Median, range	19	8–88
RPA-classification	Class IV	24	14
	Class V	79	47
	Class VI	66	39
Mental status	Normal	89	53
	Impaired	80	47
Sensorimotor deficits	No	54	32
	Yes	115	68
Steroid use	No	23	14
	Yes	146	86
MGMT promoter status	Unmethylated	76	45
	Methylated	44	26
	Unknown	49	29
Tumour location	Frontal	40	24
	Parietal	45	27
	Temporal	50	29
	Occipital	12	7
	Central	22	13
Hemisphere	Left	66	39
	Right	95	56
	Bilateral	8	5
Multilobar involvement	No	83	49
	Yes	86	51
Radiotherapy fraction dose in Gy	3.00	165	97
	2.00	1	1
	2.50	1	1
	2.67	1	1
Total Dose in Gy	Median, range	39.0	3.0–45.0
Completed radiotherapy	No	8	5
	Yes	161	95

Abbreviations: KPS—Karnofsky Performance Status; RPA—Recursive Partitioning Analysis; MGMT—O6-methylguanine-DNA methyltransferase.

## Data Availability

The datasets generated and analysed during the current study are available from the corresponding author on reasonable request.

## Abbreviations

CI: confidence interval; HR: hazard ratio; KPS: Karnofsky Performance Score; MGMT: O6-methylguanine-DNA methyltransferase; OR: odds ratio; OS: overall survival; RPA: Recursive Partitioning Analysis; RTOG: Radiation Therapy Oncology Group; WHO: World Health Organization.
